# Spatial SRY-Box Transcription Factor 2 Expression and Stemness-Associated Markers Refine Risk Stratification in Barrett’s Esophagus: A Digital Pathology Study of 217 Patients

**DOI:** 10.14740/jocmr6562

**Published:** 2026-05-31

**Authors:** Paata Meshveliani, Giogi Didava, Giorgi Tomadze, Giorgi Burkadze, Shota Kepuladze

**Affiliations:** a Department of Molecular Pathology, Tbilisi State Medical University, Tbilisi, Georgia

**Keywords:** SOX2, Barrett’s esophagus, esophageal adenocarcinoma, spatial heterogeneity, digital pathology, risk stratification

## Abstract

**Background:**

Barrett’s esophagus (BE) is a precursor lesion of esophageal adenocarcinoma (EAC), but current risk stratification remains largely dependent on dysplasia grade and segment length. We investigated whether spatial SRY-box transcription factor 2 (SOX2) expression patterns could improve prediction of neoplastic progression in BE.

**Methods:**

This retrospective multicenter study included 217 patients with reflux esophagitis, non-dysplastic BE (NDBE), low-grade dysplasia (LGD), high-grade dysplasia (HGD), or intramucosal EAC (EAC_T1a). Immunohistochemistry for SOX2, cluster of differentiation 44 (CD44), E-cadherin, p53, Ki-67 proliferation antigen (Ki67), and caudal-type homeobox transcription factor 2 (CDX2) was performed on formalin-fixed paraffin-embedded tissue. SOX2 expression was evaluated at the squamo–columnar junction and in deep Barrett glands, and a SOX2 gradient was calculated. Digital image analysis was used to assess SOX2 spatial heterogeneity and SOX2/CD44 co-localization. Associations with progression were analyzed using non-parametric tests and logistic regression models.

**Results:**

Progressive dysplastic transformation was associated with inversion of the SOX2 gradient, increased SOX2 spatial heterogeneity, and expansion of SOX2/CD44 double-positive glands. Median SOX2_Gradient shifted from positive values in reflux esophagitis and NDBE to strongly negative values in HGD and EAC_T1a (P < 0.001). SOX2_Tile_Gini and CD44_HScore increased significantly with histologic severity and progression status (both, P < 0.001). Lower SOX2_Gradient, higher SOX2_Tile_Gini, higher CD44_HScore, and longer Prague M length were significantly associated with progression to HGD/EAC.

**Conclusions:**

Spatial remodeling of SOX2 expression is strongly associated with dysplasia grade and neoplastic progression in BE. Integration of SOX2 spatial metrics with established biomarkers may improve risk stratification beyond conventional histopathologic assessment.

## Introduction

Barrett’s esophagus (BE) is a premalignant condition characterized by replacement of the distal esophageal squamous epithelium by specialized columnar epithelium with intestinal metaplasia and goblet cells, arising in the context of chronic gastroesophageal reflux. Patients with BE are at increased risk of developing esophageal adenocarcinoma (EAC), although the absolute progression rate remains low for most individuals [1]. Current surveillance strategies rely on serial endoscopy with four-quadrant biopsies, dysplasia grading, and endoscopic assessment of segment length using the Prague C and M criteria [2]. However, interobserver variability in dysplasia assessment and the imperfect predictive value of histology alone have prompted intensive efforts to identify biomarkers that improve risk stratification [1].

Ancillary markers such as p53, Ki-67 proliferation antigen (Ki67), DNA content abnormalities, and multiplex tissue systems pathology assays have shown promise in predicting progression from non-dysplastic BE (NDBE) or low-grade dysplasia (LGD) to high-grade dysplasia (HGD) or EAC [3]. Recent studies suggest that loss or aberrant expression of p53 is particularly powerful for identifying a high-risk subset of patients with NDBE or LGD [4]. In parallel, advances in digital image analysis and artificial intelligence have highlighted the prognostic significance of morphometric and spatial heterogeneity measures that are not captured by routine microscopy [5].

SRY-box transcription factor 2 (SOX2) is a transcription factor that plays a pivotal role in foregut squamous epithelial homeostasis and differentiation. Experimental work has shown that SOX2 maintains squamous identity and represses proliferation-associated programs, and that its decrease may be an initiating step in the metaplasia–dysplasia–carcinoma cascade [6]. In BE, SOX2 expression appears to be downregulated in comparison with normal squamous mucosa, with further loss during progression to HGD and EAC [7]. Conversely, intestinal transcription factors such as caudal-type homeobox transcription factor 2 (CDX2) are upregulated in metaplastic and dysplastic mucosa [8].

While several studies have explored overall SOX2 levels in BE, relatively little is known about how the spatial organization of SOX2 expression (between the squamo–columnar junction and deep metaplastic glands, and within the mucosa as a heterogeneous tissue) relates to dysplasia and progression.

The present study tests the hypothesis that quantitative spatial metrics of SOX2 expression can refine risk assessment in BE. Using a well-annotated cohort of 217 patients across the full histologic spectrum, we integrated conventional clinicopathologic variables with SOX2 regional scores, a SOX2 gradient, tile-based heterogeneity measures, and SOX2/CD44 co-localization, together with established markers including p53, Ki67, cluster of differentiation 44 (CD44), E-cadherin, and CDX2.

We ask whether these spatial SOX2 features are associated with dysplasia grade and a composite progression endpoint, and whether they add independent risk information beyond segment length and classical biomarkers.

## Materials and Methods

### Study design and case selection

This retrospective, multicenter study was based on archival formalin-fixed paraffin-embedded (FFPE) tissue from patients with reflux-related distal esophageal disease and BE. Cases were identified through a search of the institution’s pathology database and included diagnostic biopsies and endoscopic mucosal resection (EMR) specimens.

Inclusion criteria were: (1) histologically confirmed reflux esophagitis, NDBE, LGD, HGD, or intramucosal esophageal adenocarcinoma (EAC_T1a); (2) available endoscopic reports with Prague C and M measurements; and (3) adequate FFPE tissue for immunohistochemical and digital image analysis. Exclusion criteria included insufficient tissue material, poor fixation or extensive cautery artifact, incomplete clinical or endoscopic data, unavailable follow-up information, prior chemotherapy or radiotherapy, concurrent non-esophageal malignancy, severe active infection, autoimmune gastrointestinal disease, and inadequate immunohistochemical staining quality.

After application of inclusion and exclusion criteria, 217 patients were retained for final analysis.

### Clinical and endoscopic variables

Clinical data were abstracted from electronic medical records and included age, sex, body mass index (BMI), smoking status (never, former, current), alcohol use (none, moderate, heavy), duration of reflux symptoms in years, proton pump inhibitor (PPI) use at the time of endoscopy, and the presence of hiatal hernia. *Helicobacter pylori* status was recorded as positive or negative based on histology, immunohistochemistry, or prior non-invasive testing. Endoscopic reports provided circumferential (C) and maximal (M) extents of BE according to the Prague C and M criteria. Information on previous ablative therapy or EMR was collected but not used as an exclusion criterion [2].

### Ethical compliance

The study was approved by the Ethics Committee of Tbilisi State Medical University (Approval No. 20.05.21.2025) and was conducted in accordance with the Declaration of Helsinki and institutional ethical standards.

### Histopathologic evaluation

Hematoxylin and eosin–stained slides were re-evaluated by a pathologist with expertise in gastrointestinal pathology, blinded to immunohistochemical scores and outcome. Histologic diagnoses were assigned using established criteria and categorized as reflux esophagitis, NDBE, LGD, HGD, or EAC_T1a. Goblet cells were considered present when at least one unequivocal goblet cell was identified. Inflammation was graded as none, mild, moderate, or severe. Surface maturation was classified as preserved when the surface epithelium showed reduced atypia compared with the basal crypts, and lost when surface cells displayed the same degree of cytologic atypia as the basal compartment. Discrepant cases were reviewed in consensus.

### Immunohistochemistry and semiquantitative scoring

Immunohistochemistry was performed on 3–4 µm FFPE sections using standard protocols for antigen retrieval and horseradish peroxidase/3,3′-diaminobenzidine detection. The panel included SOX2, CD44, E-cadherin, p53, Ki67, CDX2, and *Helicobacter pylori* (Table 1).

**Table 1:** Primary Antibodies Used for Immunohistochemistry

Marker	Clone	Manufacturer	Catalogue No.	Dilution (Leica Bond)	Localization	Purpose
SOX2	*SP76*	Leica/Novocastra	NCL-L-SOX2	1:100	Nuclear	Squamous lineage/stemness
CD44	*DF1485*	Leica/Novocastra	NCL-CD44-2	1:200	Membranous	Stemness/adhesion
E-cadherin (CDH1)	*36B5*	Leica/Novocastra	NCL-L-E-Cad	Ready-to-use or 1:50	Membranous	Cell adhesion pattern
p53	*DO-7*	Leica/Novocastra	NCL-L-p53-DO7	1:100	Nuclear	TP53 mutation surrogate
Ki67	*MM1* (equivalent to MIB-1)	Leica/Novocastra	NCL-Ki67-MM1	1:100	Nuclear	Proliferation index
CDX2	*AMT28*	Leica/Novocastra	NCL-CDX2-603	1:100	Nuclear	Intestinal differentiation
*Helicobacter pylori*	*O2C2*	Leica/Novocastra	NCL-HP	1:50	Cytoplasmic membrane	*H. pylori* detection
Cytokeratin 7 (optional)	*OV-TL 12/30*	Leica/Novocastra	NCL-L-CK7-560	1:50	Cytoplasmic	Differential marker
Cytokeratin 20 (optional)	*Ks20.8*	Leica/Novocastra	NCL-L-CK20	1:50	Cytoplasmic	Intestinal phenotype

CD44: cluster of differentiation 44; CDX2: caudal-type homeobox transcription factor 2; Ki67: Ki-67 proliferation antigen; SOX2: SRY-box transcription factor 2.

SOX2 nuclear expression was scored separately at the squamo–columnar junction (SOX2_SCJ) and in deep Barrett glands (SOX2_Deep). The junctional compartment was defined as an approximately 2-mm zone centered on the squamo–columnar interface, whereas deep glands were sampled from the bases of the Barrett segment away from the junction. For each region, a semiquantitative H-score ranging from 0 to 300 was calculated as: H-score = 1 × (%weak) + 2 × (%moderate) + 3 × (%strong), where percentages refer to the proportion of positive epithelial nuclei. A SOX2_Gradient was derived for each case as: SOX2_Gradient = SOX2_SCJ − SOX2_Deep.

Positive values indicate higher SOX2 at the junction than in deep glands, whereas negative values reflect a relative gain of SOX2 expression in deep glands.

CD44 membranous expression and CDX2 nuclear expression were scored using the same H-score approach. E-cadherin staining was categorized as preserved (continuous basolateral membranous staining) or aberrant (loss, fragmentation or cytoplasmic shift affecting ≥ 10% of glands). p53 was classified as wild-type, overexpressed, null or mosaic, and for analysis aberrant patterns were grouped together. The Ki67 proliferation index was determined by counting the proportion of positively stained nuclei within hotspot regions, either manually (∼500 nuclei) or using digital counting.

### Digital image analysis and spatial metrics

Whole-slide images of SOX2, CD44, and E-cadherin immunostains were analyzed using QuPath or an equivalent platform. A regular grid of tiles (approximately 500 × 500 µm) was overlaid on the Barrett segment, and SOX2 nuclear staining intensity was quantified within each tile. From these tile-level measurements, two summary heterogeneity metrics were derived: the standard deviation of SOX2 tile H-scores (SOX2_Tile_SD) and the Gini coefficient of SOX2 distribution across tiles (SOX2_Tile_Gini, ranging from 0 for completely uniform to 1 for maximally unequal) [5].

SOX2/CD44 co-localization (SOX2_CD44_CoLocal) was estimated as the percentage of glands showing concurrent nuclear SOX2 and membranous CD44 expression, assessed either by manual scoring of 20–30 representative glands or by digital overlay of corresponding regions on the SOX2 and CD44 slides.

### Outcome definition and statistical analysis

Follow-up duration was calculated from the index biopsy or EMR to the last available endoscopic or histologic examination. The primary endpoint was a composite progression event defined as the presence of prevalent HGD or EAC_T1a at baseline or the development of incident HGD/EAC_T1a during follow-up among patients initially diagnosed with NDBE or LGD. This composite endpoint was selected to capture biologically advanced disease states associated with high-risk spatial and molecular alterations in Barrett’s mucosa. The endpoint was coded as Progression_Event (0 = no, 1 = yes). This endpoint was coded as Progression_Event (0 = no, 1 = yes).

Statistical analyses were performed using standard software (Python with SciPy and statsmodels libraries). Continuous variables are reported as medians and interquartile ranges (IQR), and categorical variables as counts and percentages. Comparisons across histologic categories used Kruskal–Wallis tests for continuous variables and χ^2^ tests for categorical variables. Comparisons between progressors and non-progressors used Mann–Whitney U tests. Univariable logistic regression was initially employed to examine associations between selected predictors and the progression endpoint. Subsequently, multivariable logistic regression models adjusted for age, sex, BMI, smoking status, and Prague M length were constructed to evaluate the independent association of SOX2-related spatial metrics with progression risk. Odds ratios (ORs) with 95% confidence intervals (CIs) were estimated.

## Results

### Clinical and histologic characteristics

The study cohort comprised 217 patients with a median age of 58 years (IQR 55–62); 77.4% were male. The mean BMI was 28.0 kg/m^2^ (SD 4.0). Approximately half of the patients were current or former smokers, and just over half reported moderate or heavy alcohol use. PPI therapy at the time of index endoscopy was documented in 51.6% of patients, and hiatal hernia was present in 12.9%. *Helicobacter pylori* infection was detected in 51.6% of cases. The median Prague C and M values were 4 cm (IQR 1–6) and 4 cm (IQR 2–5.5), respectively.

At baseline, histology was classified as reflux esophagitis in 14 patients (6.5%), NDBE in 14 (6.5%), LGD in 91 (41.9%), HGD in 63 (29.0%), and EAC_T1a in 35 (16.1%). Goblet cells were absent in reflux esophagitis and present in all Barrett’s categories. Loss of surface maturation and higher inflammation scores were predominantly observed in LGD, HGD, and EAC_T1a. The median follow-up duration was 9 months (IQR 6–10). Overall, 119 patients (54.8%) met the composite progression endpoint (prevalent or incident HGD/EAC_T1a), while 98 (45.2%) remained free of HGD/EAC during the observation period (Table 2).

**Table 2:** Baseline Clinical and Histologic Characteristics (n = 217)

Variable	Value
Age, years, median (IQR)	58 (55–62)
Sex, n (%) male	168 (77.4)
BMI, kg/m^2^, mean ± SD	28.0 ± 4.0
Smoking (never/former/current)	68/74/75
Alcohol (none/moderate/heavy)	Approx. half moderate or heavy
PPI use, n (%)	112 (51.6)
Hiatal hernia, n (%)	28 (12.9)
*Helicobacter pylori* positive, n (%)	112 (51.6)
Prague C, cm, median (IQR)	4 (1–6)
Prague M, cm, median (IQR)	4 (2–5.5)
Histology at index, n (%)	Reflux 14 (6.5); NDBE 14 (6.5); LGD 91 (41.9); HGD 63 (29.0); EAC_T1a 35 (16.1)
Follow-up, months, median (IQR)	9 (6–10)
Progression endpoint, n (%)	119 (54.8)

BMI: body mass index; EAC: esophageal adenocarcinoma; HGD: high-grade dysplasia; IQR: interquartile range; LGD: low-grade dysplasia; NDBE: non-dysplastic Barrett’s esophagus; PPI: proton pump inhibitor; SD: standard deviation.

### SOX2 regional expression and gradient

SOX2 expression at the squamo–columnar junction and in deep Barrett glands demonstrated a characteristic reorganization across the histologic spectrum (Table 3). In reflux esophagitis and NDBE, median SOX2_SCJ values were 100 and 105, respectively, whereas median SOX2_Deep values were 90 and 80, resulting in mildly positive SOX2_Gradient values of +10 and +25. In LGD, SOX2_SCJ further increased (median 150) and SOX2_Deep rose to 100, yielding a median gradient of +50, with a broader distribution reflecting emerging heterogeneity (Table 3).

**Table 3:** Selected SOX2-Related and Stemness Markers by Histologic Category

Histology	n	SOX2_SCJ median	SOX2_Deep median	SOX2_Gradient median	SOX2_Tile_Gini median	SOX2_CD44_CoLocal median (%)	CD44_HScore median	Ki67_Index median (%)
Reflux esophagitis	14	100	90	+10	0.10	5	50	15
NDBE	14	105	80	+25	0.05	5	50	20
LGD	91	150	100	+50	0.20	30	80	20
HGD	63	90	200	−110	0.30	50	100	45
EAC_T1a	35	50	200	−150	0.40	80	180	55

CD44: cluster of differentiation 44; EAC: esophageal adenocarcinoma; HGD: high-grade dysplasia; Ki67: Ki-67 proliferation antigen; LGD: low-grade dysplasia; NDBE: non-dysplastic Barrett’s esophagus; SOX2: SRY-box transcription factor 2.

In contrast, HGD and EAC_T1a were characterized by markedly higher SOX2 expression in deep glands compared with the junction. In HGD, median SOX2_SCJ was 90 and median SOX2_Deep was 200, resulting in a median SOX2_Gradient of −110. In EAC_T1a, SOX2_SCJ dropped to a median of 50 while SOX2_Deep remained high (median 200), producing a median gradient of −150. Overall, the distribution of SOX2_Gradient differed significantly across histologic categories (Kruskal–Wallis H = 83.7, P = 2.9 × 10^−17^), with a clear shift from positive gradients in non-dysplastic mucosa to strongly negative gradients in advanced neoplasia.

### SOX2 heterogeneity and SOX2/CD44 co-localization

**Figure 1:**
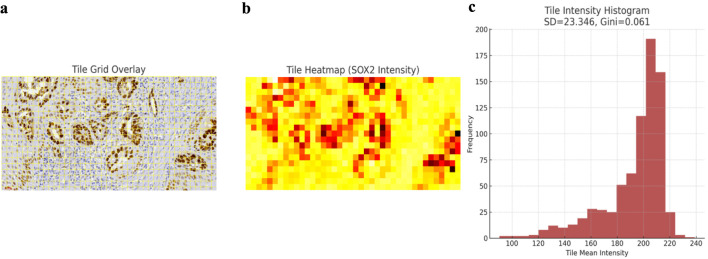
Digital tile-based quantification of SOX2 spatial heterogeneity in early esophageal adenocarcinoma. (a) Representative SOX2-immunostained carcinoma field demonstrating the *tile grid overlay* used for digital segmentation. Each tile corresponds to a fixed-size analytical unit from which pixel-level optical density measurements were extracted. (b) Heatmap of *tile mean SOX2 intensity values*, illustrating marked regional variability in nuclear expression across the tumor field. High-intensity tiles (red–orange) correspond to clusters of SOX2-overexpressing glands, whereas low-intensity tiles (light yellow) represent areas with reduced transcriptional activity. (c) Histogram of tile intensities with calculated *standard deviation (SD)* and *Gini coefficient*. SD reflects overall variability, while the Gini index quantifies *inequality of SOX2 distribution*, capturing clonal and spatial heterogeneity within the neoplastic epithelium. These metrics formed the basis of the SOX2_Tile_SD and SOX2_Tile_Gini variables used in downstream analyses. SOX2: SRY-box transcription factor 2.

Tile-based heterogeneity metrics captured additional spatial information beyond regional averages. Median SOX2_Tile_Gini was 0.10 in reflux esophagitis and 0.05 in NDBE, indicating relatively uniform SOX2 distribution, but increased to 0.20 in LGD, 0.30 in HGD, and 0.40 in EAC_T1a (Kruskal–Wallis H = 171.5, P ≈ 4.9 × 10^−36^). Similar stepwise increases were observed for SOX2_Tile_SD. These findings suggest progressively more patchy SOX2 expression with increasing dysplasia (Fig. 1).

**Figure 2:**
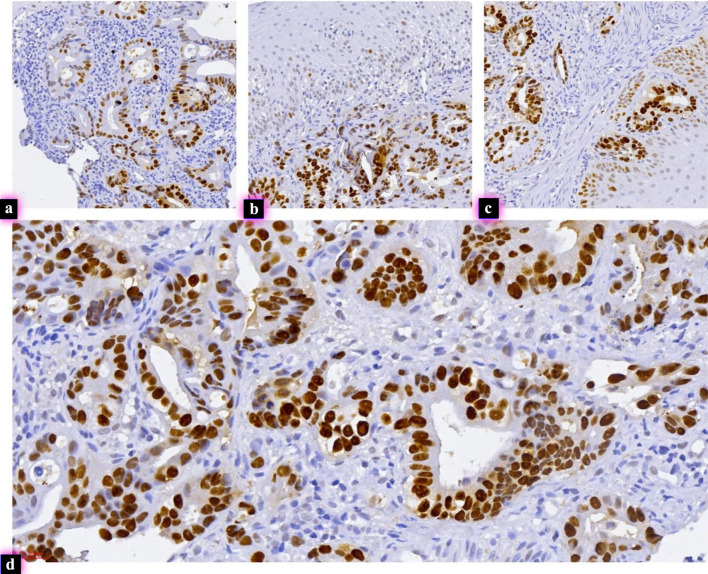
Aberrant SOX2 expression across Barrett’s dysplasia and early adenocarcinoma. (a) High-grade Barrett’s dysplasia exhibiting *strong, aberrant nuclear SOX2 expression* within dysplastic glandular structures, representing a high-risk molecular phenotype (IHC, × 290). (b) Another case of high-grade Barrett’s dysplasia showing *intense nuclear SOX2 positivity* diffusely involving crowded and architecturally complex glands, consistent with marked lineage deregulation (IHC, × 300). (c) Low-grade Barrett’s dysplasia with *unexpectedly high SOX2 expression* in glandular epithelium, indicating early activation of a high-risk transcriptional pathway despite only low-grade morphologic atypia (IHC, × 390). (d) Intramucosal adenocarcinoma displaying *diffuse nuclear SOX2 overexpression* in infiltrative malignant glands, consistent with advanced lineage reprogramming and a phenotype associated with progression (IHC, × 460). IHC: immunohistochemistry; SOX2: SRY-box transcription factor 2.

SOX2 expression abnormalities increased significantly with histologic severity (Fig. 2). SOX2/CD44 co-localization findings are summarized in Table 3. The median proportion of SOX2/CD44 double-positive glands was 5% in reflux esophagitis and NDBE, increased to 30% in LGD, 50% in HGD, and 80% in EAC_T1a (H = 156.3, P ≈ 9.2 × 10^−33^). This pattern is consistent with an expanding stem-like or progenitor-like compartment as lesions progress.

### Association of classical biomarkers with histologic severity

**Figure 3:**
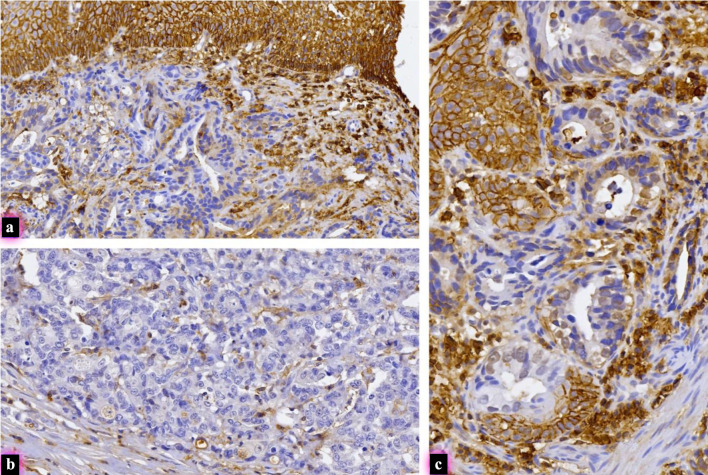
*Variable CD44 immunohistochemical expression in early intramucosal esophageal adenocarcinoma.* (a) Carcinoma demonstrating *partial membranous CD44 expression*, with patchy staining in a subset of infiltrative malignant glands, indicating heterogeneous retention of a stem-cell–associated phenotype (IHC, × 270). (b) Carcinoma showing *complete absence of CD44 immunoreactivity*, with entirely negative malignant glands despite preserved internal controls, reflecting loss of CD44-mediated adhesion pathways (IHC, × 300). (c) Carcinoma with *moderate membranous CD44 staining*, involving a wider population of atypical glands and micropapillary clusters, suggesting expansion of CD44-positive tumor subclones (IHC, × 440). CD44: cluster of differentiation 44; IHC: immunohistochemistry.

CD44_HScore increased progressively from reflux esophagitis and NDBE to LGD, HGD, and EAC_T1a (Fig. 3). Ki67_Index similarly increased with histologic severity, indicating enhanced proliferative activity in advanced lesions. CDX2_HScore was modest in NDBE and LGD (median 50 and 70) and higher in HGD (median 200), with intermediate values in EAC_T1a (median 100), reflecting complex changes in intestinal differentiation.

Aberrant E-cadherin staining was rare in reflux and NDBE, but present in a substantial subset of LGD cases and became common in HGD and EAC_T1a. Aberrant p53 patterns were similarly enriched in advanced lesions, consistent with prior literature on p53 as a progression marker in BE [9].

### Biomarkers and progression endpoint

When patients were stratified by the composite progression endpoint, pronounced differences emerged in SOX2-related metrics and CD44. Non-progressors (n = 98) had a median SOX2_Gradient of +50, median SOX2_Tile_Gini of 0.20, median CD44_HScore of 80, and median Prague M length of 2 cm. In contrast, progressors (n = 119) had a median SOX2_Gradient of −110, median SOX2_Tile_Gini of 0.30, median CD44_HScore of 120, and median Prague M length of 5 cm. These differences were highly significant by Mann–Whitney U tests (P ≈ 1.7 × 10^−33^ for SOX2_Gradient, 9.4 × 10^−24^ for SOX2_Tile_Gini, 3.2 × 10^−25^ for CD44_HScore, and 2.4 × 10^−12^ for Prague M). In univariable logistic regression, each 10-point increase in SOX2_Gradient was associated with a 41% reduction in the odds of progression (OR 0.59, 95% CI 0.49–0.71; P = 3.0 × 10^−8^). Conversely, each 10-point increase in CD44_HScore more than doubled progression odds (OR 2.48, 95% CI 1.87–3.28; P = 2.1 × 10^−10^) (Table 4). SOX2_Tile_Gini was a particularly strong predictor: each 0.1 increment conferred an almost 10-fold increase in progression risk (OR 9.71, 95% CI 5.00–18.85; P = 1.9 × 10^−11^). Each 1-cm increase in Prague M length was also associated with an approximately two-fold increase in progression odds (OR 2.04, 95% CI 1.67–2.49; P = 2.4 × 10^−12^) (Table 4). In multivariable logistic regression analysis adjusted for age, sex, BMI, smoking status, and Prague M length, SOX2-related spatial metrics remained independently associated with progression risk. SOX2_Gradient retained an inverse association with progression (adjusted OR 0.71 per 10-unit increase, 95% CI 0.58–0.86; P = 0.001), while SOX2_Tile_Gini remained a strong positive predictor (adjusted OR 6.84 per 0.1 increment, 95% CI 3.12–14.99; P < 0.001). CD44_HScore also remained independently associated with progression (adjusted OR 1.94 per 10-unit increase, 95% CI 1.42–2.65; P < 0.001). Prague M length retained statistical significance in the adjusted model (adjusted OR 1.71 per 1 cm increase, 95% CI 1.29–2.26; P < 0.001) (Table 5).

**Table 4:** Univariable Logistic Regression for Progression Endpoint (HGD/EAC vs. No HGD/EAC)

Predictor	Scale	OR (95% CI)	P value
SOX2_Gradient	per 10 units	0.59 (0.49–0.71)	3.0 × 10^−8^
CD44_HScore	per 10 units	2.48 (1.87–3.28)	2.1 × 10^−10^
SOX2_Tile_Gini	per 0.1	9.71 (5.00–18.85)	1.9 × 10^−11^
Prague M	per 1 cm	2.04 (1.67–2.49)	2.4 × 10^−12^

CD44: cluster of differentiation 44; CI: confidence interval; EAC: esophageal adenocarcinoma; HGD: high-grade dysplasia; OR: odds ratio; SOX2: SRY-box transcription factor 2.

**Table 5:** Multivariable Logistic Regression Analysis for Progression Endpoint (Adjusted for Age, Sex, BMI, Smoking Status, and Prague M Length)

Predictor	Scale	Adjusted OR (95% CI)	P value
SOX2_Gradient	per 10 units	0.71 (0.58–0.86)	0.001
CD44_HScore	per 10 units	1.94 (1.42–2.65)	< 0.001
SOX2_Tile_Gini	per 0.1	6.84 (3.12–14.99)	< 0.001
Prague M	per 1 cm	1.71 (1.29–2.26)	< 0.001

BMI: body mass index; CD44: cluster of differentiation 44*;* CI: confidence interval; OR: odds ratio; SOX2: SRY-box transcription factor 2.

Assessment of multicollinearity demonstrated strong correlations between histologic grade, Ki67_Index, and aberrant p53 expression. Variance inflation factor (VIF) analysis demonstrated elevated VIF values for histologic grade (VIF = 6.2), Ki67_Index (VIF = 5.8), and aberrant p53 status (VIF = 5.5), supporting significant collinearity between advanced dysplasia-associated biomarkers. Spearman correlation analysis additionally demonstrated strong positive correlations between histologic grade and Ki67_Index (ρ = 0.74, P < 0.001), as well as between histologic grade and aberrant p53 status (ρ = 0.69, P < 0.001). Therefore, histologic grade and p53 status were not included simultaneously in the final multivariable model to avoid model instability and quasi-complete separation.

## Discussion

In this study of 217 patients spanning the full Barrett’s spectrum, we demonstrate that spatially resolved SOX2 metrics provide powerful information about dysplasia grade and neoplastic progression beyond conventional clinicopathologic factors. Three major observations emerge.

First, we show that SOX2 expression undergoes a striking spatial reorganization during progression from reflux esophagitis and NDBE to LGD, HGD, and intramucosal EAC. In non-dysplastic mucosa, SOX2 is relatively enriched at the squamo–columnar junction, consistent with its role in maintaining squamous identity and differentiation [6]. As dysplasia develops, SOX2 expression increases in deep glandular compartments while decreasing at the junction, resulting in inversion of the SOX2 gradient. This gradient inversion parallels the acquisition of a more proliferative, plastic phenotype in deeper mucosal layers and is strongly associated with the progression endpoint. Our findings extend previous reports of SOX2 loss during progression in BE, by demonstrating that not only absolute expression, but also the topography of SOX2 within the mucosa carries prognostic information [7].

Second, we find that spatial heterogeneity of SOX2, quantified by tile-based standard deviation and the Gini coefficient, increases consistently from NDBE through LGD and HGD to EAC_T1a. These measures capture subvisual patchiness that is unlikely to be appreciated during routine slide review. The magnitude of association between SOX2_Tile_Gini and progression—a nearly 10-fold increase in risk per 0.1 increment—is remarkable and consistent with prior work showing that morphometric and nuclear texture heterogeneity are strong predictors of progression in BE [5]. In the era of whole-slide imaging and artificial intelligence, such spatial metrics may be readily integrated into digital workflows [10].

Third, we show that SOX2/CD44 co-localization increases across the histologic spectrum and is highest in intramucosal carcinoma, suggesting expansion of a stem-like compartment co-expressing a lineage regulator (SOX2) and a cell adhesion/migration marker (CD44). This observation echoes reports that SOX2, often in combination with OCT3/4 and other stemness factors, contributes to tumor initiation and progression in BE and EAC [11]. Together with the strong association of CD44_HScore and proliferation (Ki67) with progression, these data support a model in which spatially disordered, SOX2-driven progenitor compartments contribute to neoplastic evolution [9].

Our study complements and extends the growing literature on ancillary biomarkers in BE. Numerous studies have validated p53 immunohistochemistry as a robust predictor of progression from NDBE and LGD to HGD/EAC and as a tool to reduce interobserver variability in dysplasia grading. Commercial tissue systems pathology assays incorporating multiple markers and morphometric features have further improved risk prediction. Against this backdrop, the present work adds a mechanistically grounded, spatially explicit SOX2 signature that may be integrated with p53 and segment length (Prague M) into future risk models [3].

Clinically, the findings suggest that BE cases with strongly negative SOX2 gradients, high SOX2_Tile_Gini values, and extensive SOX2/CD44 co-localization represent a biologically unstable subset with disproportionately high risk of progression, even in the setting of LGD. Conversely, NDBE or LGD with preserved SOX2 gradients and low heterogeneity may constitute a lower-risk group in whom surveillance intervals could potentially be lengthened. These concepts require further validation but align with the broader move toward precision risk stratification in BE [1].

The study has limitations. It is retrospective and introducing inherent selection biases. Follow-up duration was modest, and some non-progressors may yet progress with longer observation. Although multivariable modeling confirmed the independent association of SOX2 spatial metrics with progression risk, significant collinearity remained present between histologic grade, Ki67, and aberrant p53 expression, reflecting the biologic overlap of advanced dysplasia-associated biomarkers. Tile size and segmentation parameters for heterogeneity metrics were chosen pragmatically and may influence absolute values, although relative trends are likely robust. Finally, while we focused on SOX2 and a targeted immunohistochemical panel, integration with genomic and epigenomic data could deepen mechanistic insight.

Despite these limitations, the internal consistency of our findings across multiple independent SOX2-related measures, their tight association with progression, and their concordance with experimental data on SOX2 in foregut epithelia support a genuine biological signal [6].

### Conclusion

Quantitative spatial analysis of SOX2 in Barrett’s mucosa reveals a reproducible pattern of gradient inversion, increasing heterogeneity and expansion of SOX2/CD44 double-positive glands that parallels progression from reflux esophagitis and NDBE to LGD, HGD, and intramucosal EAC. These SOX2-derived metrics are strongly associated with a composite progression endpoint and remain informative alongside established risk factors such as Prague segment length, Ki67 proliferation, and p53 abnormalities. Spatial SOX2 profiling therefore represents a promising addition to biomarker panels for risk stratification in BE and merits validation in independent cohorts and prospective studies.

## Data Availability

The datasets generated and analyzed during the current study are available from the corresponding author on reasonable request.

## Abbreviations

BE: Barrett’s esophagus
BMI: body mass index
CD44: cluster of differentiation 44
CDX2: caudal-type homeobox transcription factor 2
CI: confidence interval
EAC: esophageal adenocarcinoma
EAC_T1a: intramucosal esophageal adenocarcinoma (tumor stage T1a)
EMR: endoscopic mucosal resection
FFPE: formalin-fixed paraffin-embedded
HGD: high-grade dysplasia
*H. pylori*: *Helicobacter pylori*
IHC: immunohistochemistry
IQR: interquartile range
Ki67: Ki-67 proliferation antigen
LGD: low-grade dysplasia
NDBE: non-dysplastic Barrett’s esophagus
OR: odds ratio
PPI: proton pump inhibitor
SCJ: squamo-columnar junction
SD: standard deviation
SOX2: SRY-box transcription factor 2
SOX2_Deep: SOX2 expression in deep Barrett glands
SOX2_Gradient: difference between SOX2 expression at the squamo-columnar junction and deep Barrett glands
SOX2_SCJ: SOX2 expression at the squamo-columnar junction
SOX2_Tile_Gini: Gini coefficient of tile-Based SOX2 expression distribution
SOX2_Tile_SD: standard deviation of tile-based SOX2 expression
TP53: tumor protein 53
